# Supplementary material for: Functional Impact of BeKm-1, a High-Affinity hERG Blocker, on Cardiomyocytes Derived from Human-Induced Pluripotent Stem Cells
Source: Int J Mol Sci. 2020 Sep 28;21(19):7167. doi: 10.3390/ijms21197167 (PMC7582727; doi:10.3390/ijms21197167)
Supplement: Supplementary file 1 [file ijms-21-07167-s001.pdf]

Supplementary data

Functional impact of BeKm-1, a high affinity hERG blocker, on cardiomyocytes derived from human-induced pluripotent stem cells

Introduction

The $K_V11.1$ channel presents peculiar features compared to other voltage-dependent potassium channels showing a slow activation (in the order of a second) and a fast inactivation (within the millisecond scale) [1]. As a result, the activation gate of the channel opens gradually during the plateau phase of the action potential (AP), while the channel remains at least partially inactivated. When repolarization starts, the functional availability of $K_V11.1$ channel progressively grows due to fast recovery from inactivation, resulting in an outward current that largely compensates for the decrease in the driving force as the membrane potential gets closer to the equilibrium potential for potassium ions. These properties define hERG as a major component of cardiac repolarization, both in atrial and ventricular cells (**Supplementary Figure S1**).

References

1. Bett, G.C.; Zhou, Q.; Rasmusson, R.L. Models of HERG gating. *Biophys J* **2011**, *101*, 631-642, doi:10.1016/j.bpj.2011.06.050.
2. O'Hara, T.; Virag, L.; Varro, A.; Rudy, Y. Simulation of the undiseased human cardiac ventricular action potential: model formulation and experimental validation. *PLoS Comput Biol* **2011**, *7*, e1002061, doi:10.1371/journal.pcbi.1002061.

Supplementary figures

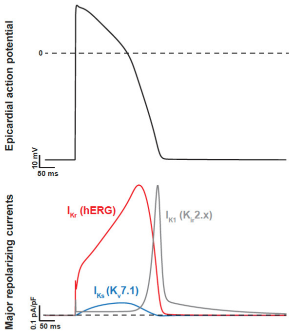

Figure S1. Representation of the epicardial AP and the major repolarizing currents [2]. Dotted lines = 0 mV or pA. Scale bars: horizontal = 50 ms, vertical = 10 mV / 0.1 pA/pF.

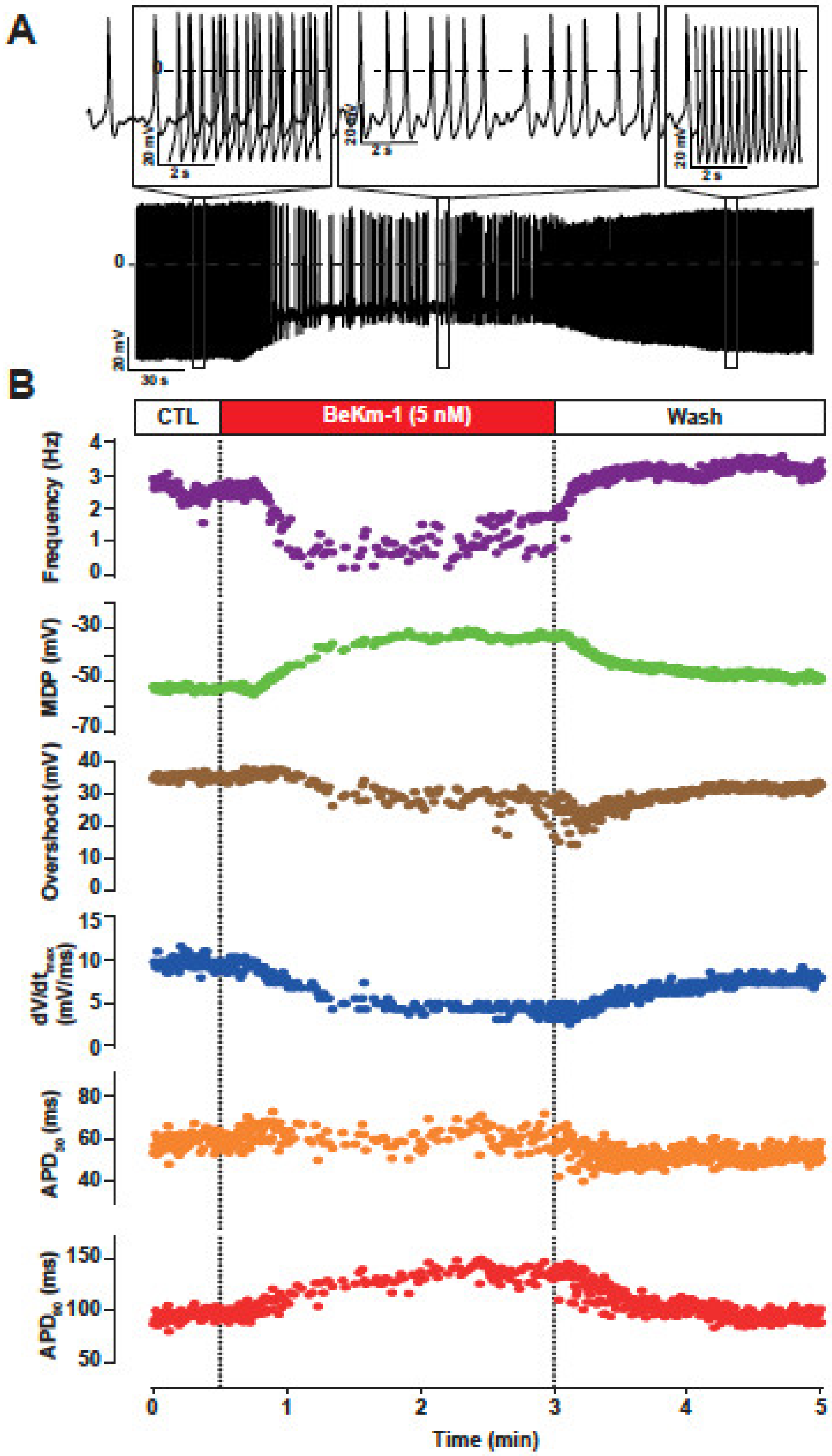

Figure S2. Kinetics of 5 nM BeKm-1 application and toxin wash effects on spontaneous APs of hiPS-CMs. (A) Bottom: representative raw trace from manual patch-clamp recording of spontaneous APs on a ventricular-like cardiomyocyte. Top: magnification of this trace focusing on vehicle (CTL, left), 5 nM BeKm-1 application (middle) and wash (right) effects on APs. (B) Representative time courses of 5 nM BeKm-1 application and toxin wash corresponding to parameters from APs depicted in panel (A). AP parameters analyzed are from top to bottom: frequency, MDP, overshoot, maximal upstroke velocity, APD_{30} and APD_{90}. The corresponding

treatments during recording are depicted on top (i.e. CTL, BeKm-1 and wash) and aligned to the raw trace in (A) and the time courses below where they are separated by dotted lines.

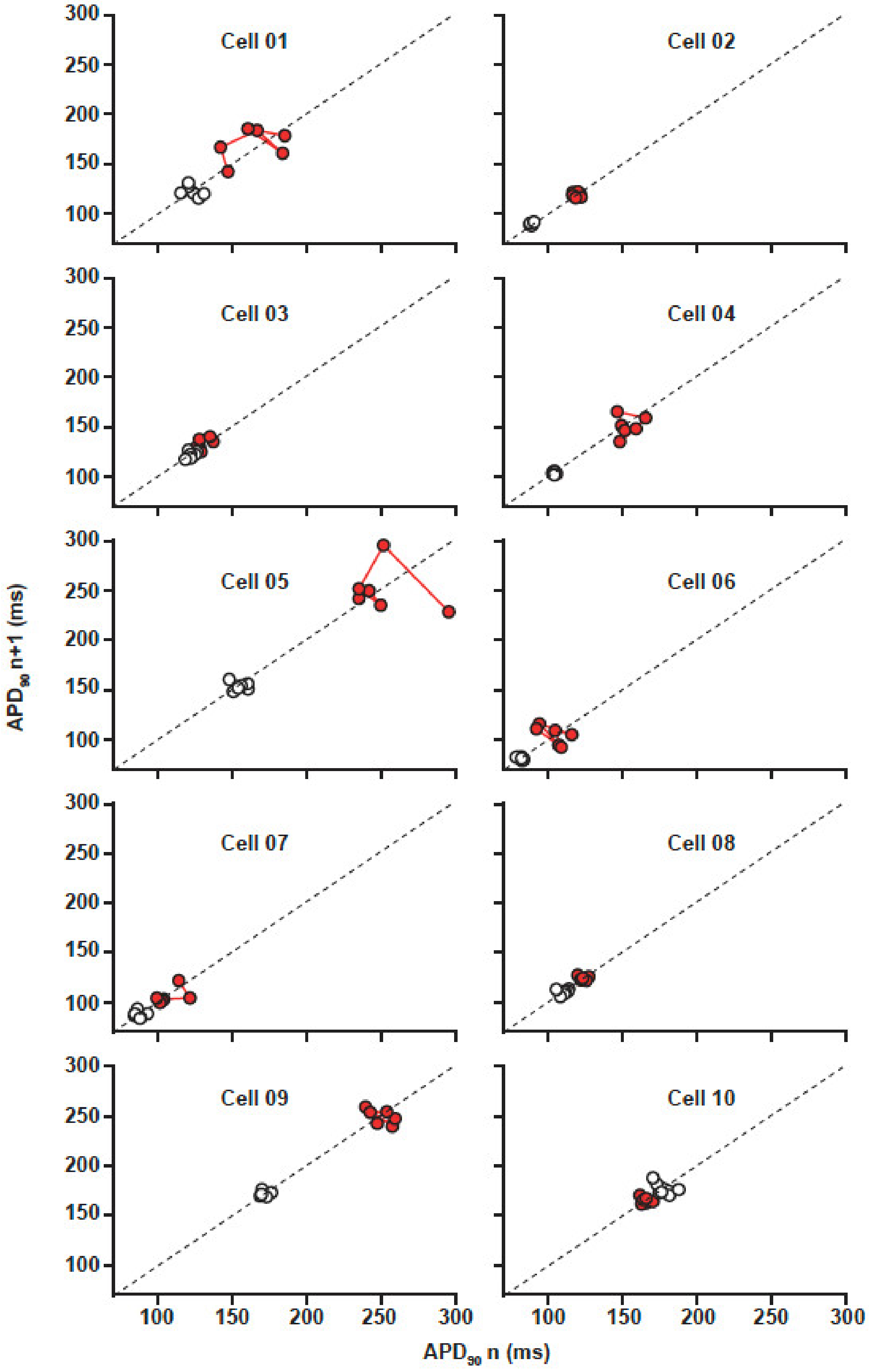

Figure S3. Box plot representation of APD_{90} change by 5 nM BeKm-1 for all ventricular hiPS-CMs recorded in the dynamic patch-clamp method.

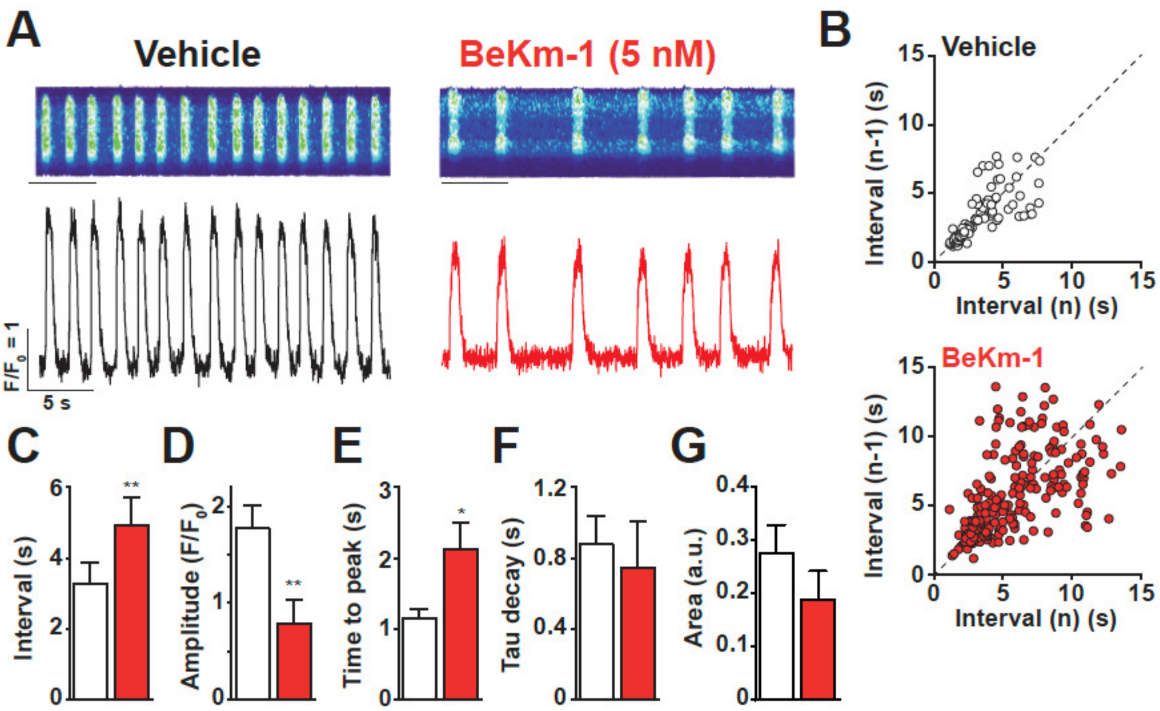

Figure S4. BeKm-1 affects the shape and frequency of intracellular CaT in hiPS-CMs. (A) Representative kymograph (top) and CaT (bottom) before (black trace) and with 5 nM BeKm-1 application (red trace). (B) Poincaré plot of CaT interval dispersion before (top) and with 5 nM BeKm-1 application (bottom). (C-F) Average interval duration between CaT (C), amplitude (F/F_0) (D), time to peak (E) and tau of decay (F) in both conditions. n = 9 hiPS-CMs with more than 10 CaT averaged for each cell. Paired t-tests with *, p<0.05; **, p<0.01.

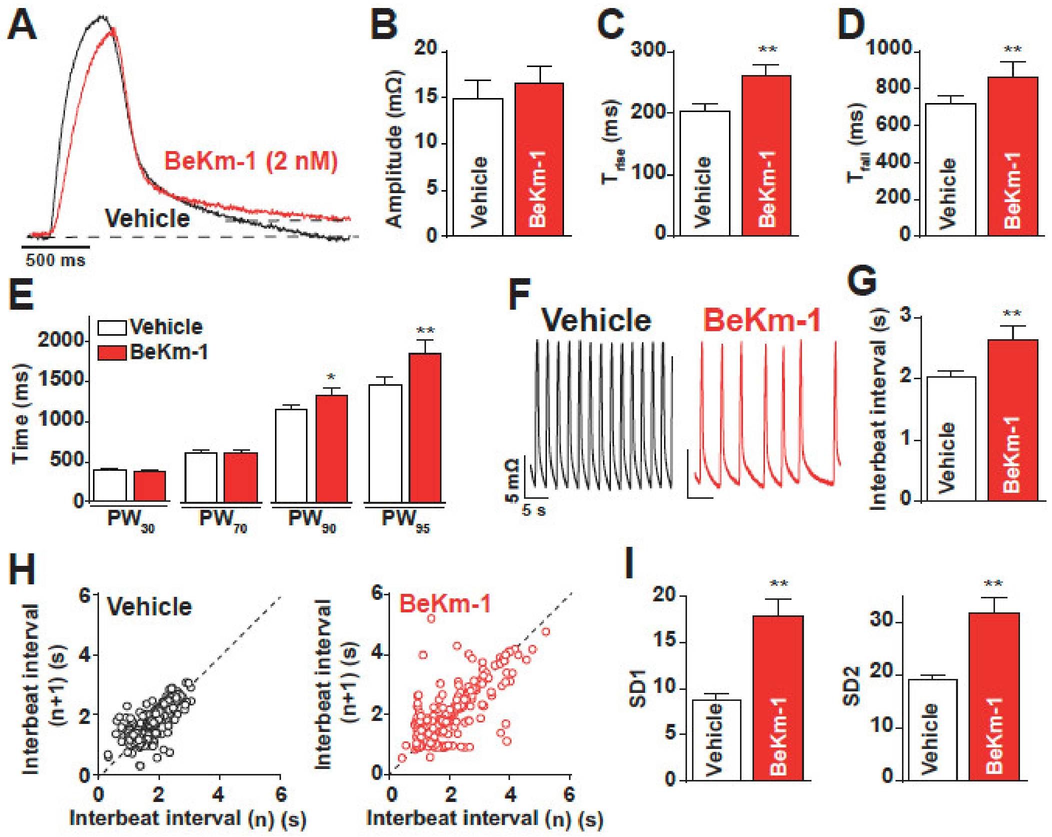

Figure S5. BeKm-1 changes contractile parameters in hiPS-CMs. (A) Superimposed traces of representative hiPS-CMs monolayers contractions before and after application of 2 nM BeKm-1. Traces were normalized to peak amplitude. Upper dotted line shows the position of PW_{95}. (B-E) Average effects of 2 nM BeKm-1 on contraction amplitude (B), contraction rising time (T_{rise}) (C), relaxation time (T_{fall}) (D) and PW at 30%, 70%, 90% and 95% relaxation times (PW_{30}, PW_{70}, PW_{90} and PW_{95}, respectively) (E). (F) Representative contraction traces from hiPS-CMs monolayers before (left panel) and after (right panel) application of 2 nM BeKm-1 illustrating rhythm differences induced by the toxin. (G) Average interbeat interval in both conditions. (H) Plot of interbeat intervals from consecutive contractions in vehicle condition (left panel) and after application of 2 nM BeKm-1 (right panel). (I) Average variation of the mean SD1 (standard deviation of instantaneous contraction-to-contraction interval variability) and SD2 (standard deviation of continuous long-term interval variability) in both conditions. n = 8 independent hiPS-CMs monolayers (10 sweeps of 30 seconds for each) analyzed for each condition. Paired t-tests with *, $p<0.05$ and **, $p<0.01$.

A

Cell type	Ventricular	Atrial	Nodal
APD_{50}/APD_{90}	x > 0.7	x < 0.7	
Mean dV/dt $_{max}$ (mV/ms)	x > 10	10 > x > 3	x < 3
Mean amplitude (mV)	x > 90	90 > x > 75	x < 75
MDP (mV)	x < -50	-50 < x < -45	x > -45

B

Parameter	Mean value	SD	n
APD_{50}/APD_{90}	0.739479033	0.0407736	6
Mean dV/dt $_{max}$ (mV/ms)	13.8877848	11.0357205	
Mean amplitude (mV)	85.0663681	10.5289962	
MDP (mV)	-53.2510663	3.90286656	

C

Parameter	Mean value	SD	n
APD_{50}/APD_{90}	0,691916339	0,0604766	10
Mean dV/dt $_{max}$ (mV/ms)	191,3948726	67,417927	
Mean amplitude (mV)	136,0553688	12,8810274	
MDP (mV)	-81,22966777	3,14683623	

Table S1. (A) List of AP parameters used for hiPS-CMs subtype classification. (B) Mean values for recorded spontaneous ventricular APs. (C) Mean values for recorded dynamically clamped APs.
